# Mapping the Vulnerability of Older-Adult Neighborhoods: An Ecological Study of New York State

**DOI:** 10.3390/ijerph22030332

**Published:** 2025-02-24

**Authors:** Samantha Friedman, Chunxu Fang, Tse-Chuan Yang, Rui Li, Imran Hossain Mithu, Jennifer A. Manganello, Xiaobo Romeiko, Shao Lin

**Affiliations:** 1 Department of Sociology, University at Albany, State University of New York, 348 Arts & Sciences Building 1400 Washington Avenue, Albany, NY 12222, USA; samfriedman@albany.edu (S.F.); cfang2@albany.edu (C.F.); tyang3@albany.edu (T.-C.Y.); 2 Department of Geography and Planning, University at Albany, State University of New York, 348 Arts & Sciences Building 1400 Washington Avenue, Albany, NY 12222, USA; rli4@albany.edu; 3 Community, Environment and Policy Division, Mel and Enid Zuckerman College of Public Health, University of Arizona, Tucson, AZ 85721, USA; imranmithu@arizona.edu; 4 Department of Health Policy, Management, and Behavior, College of Integrated Health Sciences, University at Albany, State University of New York, 1 University Place, Rensselaer, NY 12144, USA; jmanganello@albany.edu; 5 Department of Environmental Health Sciences, College of Integrated Health Sciences, University at Albany, State University of New York, 1 University Place, Rensselaer, NY 12144, USA; xxue@albany.edu; 6 Department of Epidemiology and Biostatistics, College of Integrated Health Sciences, University at Albany, State University of New York, 1 University Place, Rensselaer, NY 12144, USA

**Keywords:** older-adult neighborhoods, demographic, economic, and social inequality, vulnerability of aging communities, New York State

## Abstract

We examined neighborhood-level demographic, economic, and social characteristics and food and health-services access to gauge the vulnerability of older-adult neighborhoods in New York State (NYS), which is understudied and is significant given the rapid aging of populations worldwide. We conducted descriptive ecological analyses using data from the American Community Survey, historical redlining maps, Social Capital Instruments, U.S. Department of Agriculture food access atlas, ESRI businesses, and Social Determinants of Health. We compared census tracts classified as having high and low levels of older-adult population; among those identified as high-older-adult neighborhoods, we then examined tracts with high and low levels of adult population living alone and in poverty. Our results showed that NYS neighborhoods with large shares of the older adult population are generally faring well in terms of their socioeconomic status, social capital, lack of social isolation, and health services access. However, the older-adult neighborhoods with larger shares of the population living alone and in poverty fare worse, living in areas with poorer socioeconomic status, lower social capital, and considered medically underserved. NYS older adult communities are projected to increase by 2030. Resources should be invested in such areas with vulnerable groups so populations may age in equitable and accessible communities.

## 1. Introduction

New York State (NYS) ranks fourth in the nation in terms of the number of people aged 60 and over, with 4.6 million adults falling into this older-age range [1]. By 2030, the number of counties with at least 30% of its population falling into this older-age range will increase to 33, up from only 9 counties in 2020, which means that more than half of NYS’s 62 counties will be composed of significant shares of older adults [1]. Given that many counties will have increasing shares of older adults, it is important to examine the aging population in NYS at the neighborhood level to document the characteristics of their immediate residential environments.

NYS encompasses neighborhoods spanning a wide variety of geographic contexts, including very densely populated urban areas like New York City, small-to-mid-size cities like Albany and Syracuse, suburban areas in Westchester County, and rural areas in the Adirondacks and Finger Lakes region. It is a microcosm of different contexts that are seen within the larger United States (U.S.) that vary with respect to their demographic composition, economic resources, community networks and social isolation, and food and health services access and is, therefore, an ideal study site to examine aging communities. The NYS government recognized the significance of the diversity in its communities and divided the state into 10 separate economic regions to foster and enhance economic development tailored to each area [2].

The aging of the population in NYS mirrors the increase in older adults worldwide, where in many countries, this segment of the population is aging at a faster pace than in earlier decades [3]. Recognizing the challenges faced by countries and local communities in fostering the healthy aging of the population, the United Nations (UN) endorsed an action plan in 2020 called the “UN Decade of Health Ageing 2021–2030,” which mandates a collaboration between the World Health Organization (WHO) and the UN for 10 years to work to improve the lives of older adults and their communities [3]. This work builds upon the earlier work of the WHO to build “a global network of age-friendly cities and communities (AFCC)” [4]. The main principle underlying the establishment of AFCC is the optimization of “opportunities for health, participation and security in order to enhance quality of life as people age” [4]. The WHO proposed eight domains of life in which challenges would be faced by cities with aging populations and thus in which actions are needed [4]. However, in a later 2015 report, the WHO developed “core indicators” of AFCC in three areas—(1) equity, (2) an accessible physical environment, and (3) an inclusive social environment [5]. Most of the work gauging the age-friendliness of cities and communities has focused on cities rather than neighborhoods, per se, although research in the U.S. is surprisingly quite limited [6].

Against this backdrop of AFCCs, a growing literature specifically assesses the vulnerability of older adult communities at the neighborhood level in order to document the potential needs of these areas [7,8,9]. This latter set of studies builds upon a larger literature examining the social vulnerability of all neighborhoods and not just those restricted to communities with larger shares of older-adult populations or what we refer to as “older-adult neighborhoods” [10,11,12,13,14]. Social vulnerabilities are characteristics of communities like poverty, lower education, weak social ties and social isolation, and poor health that make it difficult for communities to withstand external stressors like climate change, economic downturns, or health crises. A study in Canada, for example, finds that urban neighborhoods that have high proportions of older adults are more likely to have higher levels of poor and foreign-born residents [7].

By identifying the characteristics of older-adult neighborhoods that make them potentially vulnerable in terms of demographic, economic, and social factors and food and health services access, policymakers and community planners can develop targeted strategies to enhance the quality of life for this growing population segment, consistent with the goal of AFCCs and the UN Decade of Ageing. Despite the graying of NYS’s communities, no research, to our knowledge, has documented the vulnerability of older-adult neighborhoods in NYS.

Our study fills this significant gap by providing an ecological portrait of the vulnerability of census tracts in NYS. Our main objective is to examine whether census-tract level outcomes, including demographic, economic, and social characteristics; community networks and social isolation; and food and health access vary by the nature of the older-adult population in these neighborhoods. This objective is critical to add to the limited but growing literature that evaluates the core indicators of AFCC under the WHO Health Ageing Initiative. Very little research examines these indicators at the census tract level within the US [6]. Specifically, we make three types of comparisons of these characteristics between census tracts classified as (1) older adult versus not older adult; and among tracts classified as older adult, we compare tracts identified (2) as those with large shares of the population living alone versus not living alone; and (3) as those with high levels of poverty versus low levels of poverty.

## 2. Materials and Methods

We conducted descriptive ecological analyses using data from the American Community Survey (ACS), historical redlining maps, Social Capital Instruments, U.S. Department of Agriculture food access atlas, Modified Retail Food Environment Index, ESRI businesses, and Social Determinants of Health. These data are all publicly available, and we obtained them from publicly available websites (see the links to the data in the data availability statement below). Because we rely on secondary data sources, there are no human subjects in this study.

We chose these data sources because they are the best available data and most accurate to measure demographic, economic, and social characteristics; community networks and social isolation; and food and health access across census tracts in NYS. As discussed in more detail below, all data except the data on Social Capital Instruments and ESRI business data are from government sources (i.e., U.S. Census Bureau, U.S. Department of Agriculture, the Centers for Disease Control and Prevention and the Agency for Healthcare Research and Quality), thereby ensuring that the data are of the highest quality. For decades, ESRI has been a leader in providing data on businesses [15]. The data on Social Capital Instruments has been used by other researchers, including Yang et al. [16], even though there is not necessarily a consensus on how to measure social capital.

Our analyses were conducted using all census tracts in NYS; we did not exclude any areas. The primary focus of our descriptive ecological analyses is making comparisons of two groups of census tracts deemed as “high” and “low” in terms of three characteristics—(1) the percentage of the population aged 65 and older (i.e., “older-adult population”); (2) the percentage of the older-adult population living alone; and (3) the percentage of the older-adult population living in poverty. As we discuss in more detail below as we go into depth about each data source, we use cutoffs for these three variables at the 75th percentile to distinguish between “high” and “low” values, consistent with other research [17].

The primary data we use to classify census tracts in NYS as “older adult” come from the ACS 2019, 5-year data [18]. Tracts classified in the “high” older-adult group are at or above the 75th percentile of percentage of adults aged 65 and older, which is 19.81%; tracts classified as in the “low” category fall below the 75th percentile (i.e., less than 19.81%). The number of census tracts in this category in NYS is 1213. We then disaggregate census tracts classified as “high” in terms of their population being aged 65 and older (i.e., n = 1213), based upon their percentage: (a) living alone and (b) falling below the poverty line. For that set of classifications, we also use the 75th percentile as the threshold to distinguish between tracts in the “high” and “low” ranges of percentage of older adults living alone and below poverty. The 75th percentile values for the census tract percentages living alone and in poverty are 36.03% and 17.14%, respectively, and the number of census tracts in these “high” categories are 316 and 156, respectively.

We characterize older-age communities based on demographic and socioeconomic factors as well as their social and physical environments, drawing from a number of data sources. Census tract-level data on education, income, homeownership, disability, unemployment, racial and ethnic composition, and age composition are sourced from 2019 ACS. Additionally, the same data provide variables used to calculate the social isolation index, an indicator of the social environment of neighborhoods, including (1) poverty, (2) living alone, (3) divorced, separated, or widowed status, (4) never married status, (5) disability, and (6) difficulty with independent living. Following previous reports [19], the risk of social isolation in a census tract is measured by the percentile of average z-scores for these six social isolation-related risk factors. The percentile is calculated based on all census tracts in NYS with complete information on these variables.

The redlining score, which is an indicator of the historical mortgage investment level in neighborhoods, is calculated by overlaying the current census tract boundaries with historical redlining maps from the Mapping Inequality project [20]. The methodology for calculating the HOLC (Home Owners’ Loan Corporation) score is derived from the National Community Reinvestment Coalition (NCRC) [21]. For each census tract, we first calculate the percentage of the area that overlaps with the four redlining map categories: ‘A = Best’, ‘B = Still Desirable’, ‘C = Definitely Declining’, and ‘D = Hazardous’. These percentages are then multiplied by their corresponding numerical values (1–4) representing the four grades. The weighted average of these values is used to determine the HOLC grade. A score of 4.0 indicates that the entire census tract overlaps with an area marked as ‘D = Hazardous’ on the redlining map.

The 2019 Food Access Research Atlas provides numerous variables measuring accessibility to healthy food and neighborhood-level indicators of resources, such as average income level. For a census tract, “low access to healthy food is defined as a significant number (at least 500 people) or share (at least 33 percent) of the population being far from a supermarket, supercenter, or large grocery store” [22]. The definition of ‘being far’ varies: our analysis includes distances greater than (1) one-half mile for urban areas or greater than ten miles for rural areas, (2) one mile for urban areas or greater than ten miles for rural areas, and (3) more than twenty miles regardless of vehicle availability. For example, a low-access tract measured at 1 mile and 10 miles indicates the number of tracts that live more than 1 mile (urban areas) or more than 10 miles (rural areas) from the nearest supermarket, supercenter, or large grocery store [22].

The Modified Retail Food Environment Index (mRFEI) is derived from the Children’s Food Environment State Indicator Report released in 2011 by the CDC’s Division of Nutrition, Physical Activity, and Obesity. This variable indicates the percentage of healthy food retailers within each census tract. A lower mRFEI may suggest a low density of healthy food retailers, such as supermarkets, or a high density of unhealthy food retailers, such as fast food restaurants [23].

Health services data are obtained from two sources—from ESRI business analyst data via ArcGIS Pro and the Social Determinants of Health (SDOH) database compiled by the Agency for Healthcare Research and Quality (AHRQ). ESRI business analyst data include business and points of interest data [24]. Based upon these data, within each census tract in NYS, we analyzed the number of health services and then examined the number as a percentage of all businesses. From those data, for each census tract, we also calculated the total household spending on health services, including insurance expenditures.

From the AHRQ SDOH data, we use two different measures to gauge the health services geography in NYS census tracts [25]. We use an indicator of whether the tract is considered to be a medically underserved area (MUA), which are areas that lack medical services. Census tracts obtain a score based upon four characteristics of their area: (1) medical provider per 1000 population ratio; (2) percent of the population in poverty; (3) percent of the population aged 65 and older; and (4) the infant mortality rate. Tracts with scores at or below a threshold of 62 qualify as an MUA. We also use the average distance calculated from the centroids of each census tract to the nearest health service facility of each of the following types: (1) emergency department; (2) medical-surgical intensive care unit; (3) designated trauma center; (4) health clinic; and (5) hospital with alcohol and drug abuse inpatient care.

Social capital, which is a measure of the social environment, is categorized into three types: bonding capital, bridging capital, and linking capital. Bonding capital is calculated based on nine indicators that measure the similarity among community residents, including age, race, class, gender, language, and community capacity. Bridging capital assesses how well residents, through their membership in various associations, can connect different segments of society. This is measured by the number of unions and religious, civic, social advocacy, and charitable organizations, as well as the percentage of residents participating in fraternal orders. Linking capital captures political connections at the local, state, and federal levels and measures political activities. The overall social capital index for each census tract is the average of these three subindices. We obtained these measures from Fraser and colleagues [26], who, in their publication, provide more details about the methodology they used to construct these measures. On all of these measures, higher values indicate greater levels of social capital.

In terms of the methods for analysis, for between-group comparisons, if the dependent variable of interest is continuous, the Welch Two Sample *t*-test is used. This test is similar to the *t*-test but does not require the assumption of equal variance between populations. If the outcome of interest is a categorical variable, we use the chi-square test to determine whether there are significant differences between high-older-adult tracts and low-older-adult tracts. The only exception is when the number of census tracts in a subgroup is too small; in such cases, Fisher’s exact test is used. Notes under the tables indicate which test was used to generate the *p*-value.

## 3. Results

### 3.1. Spatial Variation in Older-Adult Neighborhoods

**Figure 1:**
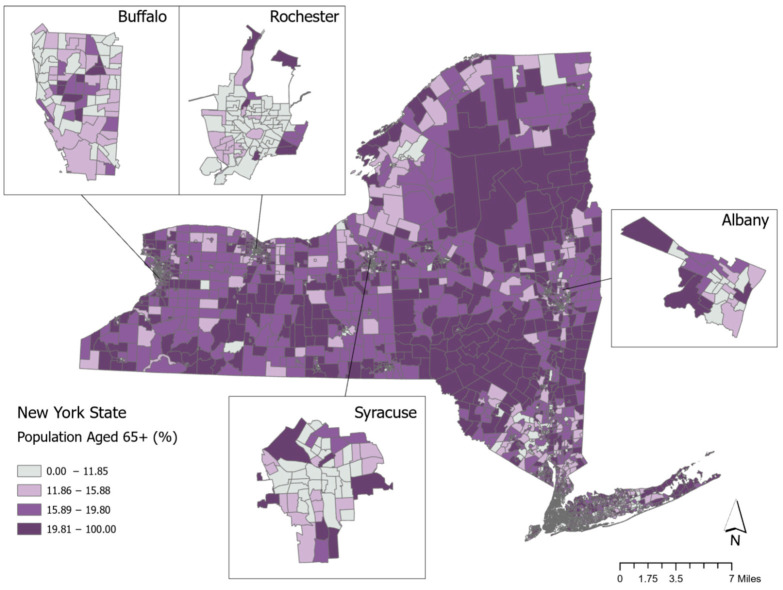
Map of percentage of the population 65 years of age and over in New York State.

Figure 1 shows the variation across NYS census tracts in the percentage of the population over 65 years of age. The median percentage is 15.89%, and the value at the 75th percentile is 19.81%, which is shaded in the darkest purple category. We consider values at the 75th percentile and higher to be neighborhoods with the highest level of older adult population. Figure 1 shows the distribution of older-adult neighborhoods across the entire state and provides insets of the four largest upstate cities of Buffalo, Rochester, Syracuse, and Albany. The overall NYS map shows that many of the census tracts with the highest levels of population of adults aged 65 and older are in tracts that are located outside of the main metropolitan areas that are in the Catskill and Adirondack Mountain regions as well as in central parts of Western New York in the Finger Lakes region. In the four upstate city maps, the census tracts with the largest shares of older adult populations are located on the outskirts of the cities rather than in the downtown areas.

**Figure 2:**
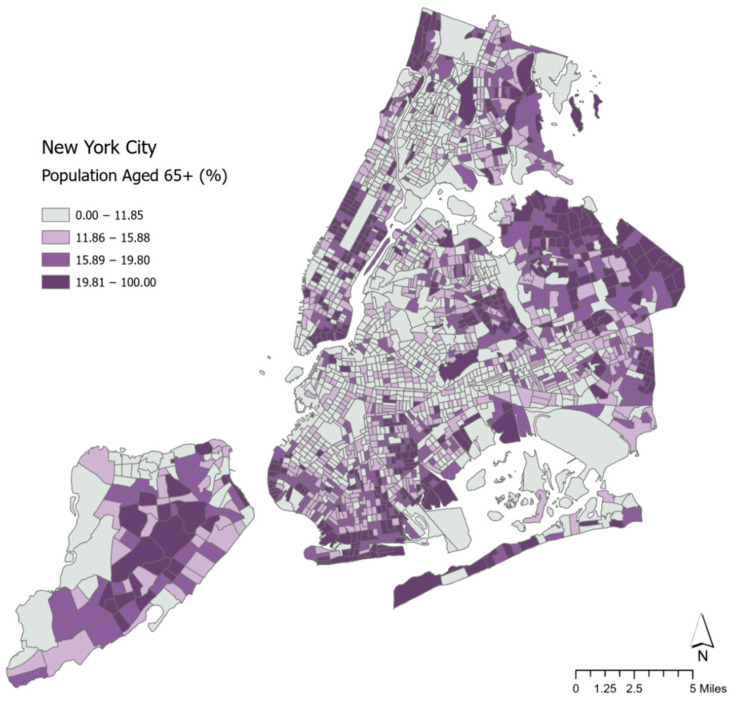
Map of percentage of the population 65 years of age and over in New York City.

Figure 2 focuses specifically on census tracts within New York City. In Manhattan, the neighborhoods with the largest shares of the older adult population are the Upper East and West Side areas, as well as the Lower East Side. In the other boroughs, the census tracts with percentages of the older population at or above the 75th percentile are located in eastern Queens, the Rockaways, Southern Brooklyn, central Staten Island, and the upper western part of the Bronx, as well as some eastern areas of the Bronx.

### 3.2. Demographic and Economic Characteristics of Older-Adult Neighborhoods

We now focus on analyzing the demographic and socioeconomic characteristics of these neighborhoods that we have visualized, as well as neighborhoods that are subdivided based on the percentage of older adults living alone and separately for those living in poverty. Table 1 reports these descriptive analyses. Columns 1 and 2 of Table 1 compare characteristics of census tracts that fall in the “High” range or 75th percentile of neighborhoods in which the percentage of the population in the 65+ age range is at least 19.81% to census tracts that fall in the “Low” range or those with a percentage of the population aged 65+ that is below 19.81%. The socioeconomic and demographic characteristics of high-older-adult census tracts are significantly better than those in low-older-adult census tracts. In high-older-adult tracts, an average of 40% of the population has at least a college degree, which is seven percentage points higher than the average of 33% in the low-older-adult census tracts. This difference is statistically significant. In high-older-adult census tracts, the median household income and homeownership rates are also significantly higher than in low-older-adult tracts. The average percentages of the population who are disabled and unemployed are significantly lower in high-older-adult census tracts than in neighborhoods with fewer older adults.

Columns 5 through 12 of Table 1 focus on the high-older-adult census tracts (*n* = 1213) and disaggregate them separately by the percentage living alone (columns 5 through 8) and the percentage living below the poverty line (columns 9 through 12). “High” groupings within these variables are at or above the 75th percentile values for each of these two variables. With respect to the differences in socioeconomic and demographic characteristics in census tracts characterized by a “high” population of those living alone versus those as “low,” they emerge for homeownership and disability rates. High-older-adult living alone census tracts have an average homeownership rate that is significantly lower than low-older-adult living alone census tracts (56% versus 77%, respectively). On the other hand, high-older-adult living alone census tracts have significantly greater shares of households with disabled persons than those living in low-older-adult living alone areas (37% versus 30%).

The disparities between tracts with older adults that have poverty rates at or above the 75th percentile compared to those with poverty rates below the 75th percentile level follow a similar pattern (see columns 9 through 12). Tracts with older adults who are the poorest (in the “high” range) have higher levels of economic insecurity than those with older adults who have fewer poor people (in the “low” range). Column 9 shows that in the high group of older adults living below the poverty line, an average of 34% have at least a bachelor’s degree, relative to 41% of those in tracts that have a low level of older adults living in poverty. Within census tracts designated as high-older-adult areas in poverty, the average median income and homeownership rate are significantly lower than those in census tracts designated as low in terms of older adult areas in poverty (see columns 9 and 10). The average percentage of persons with disabilities or the unemployment rate is significantly higher in high-older-adult poverty tracts than in low-older-adult poverty tracts.

### 3.3. The Racial/Ethnic Composition and Redlining Status of Older-Adult Neighborhoods

How racially and ethnically diverse are NYS older-adult neighborhoods? Columns 1 to 4 of Panel A in Table 2 tackle this question. The data in column 1 of Table 2 show that on average, 73% of the racial and ethnic composition in high-older-adult neighborhoods, or those with the percentage of older adults at the 75th percentile and above, is composed of non-Hispanic Whites (hereafter “Whites”). However, when we focus on the data in column 2, there is more racial and ethnic diversity in NYS census tracts that have lower shares of older adults (i.e., low-older-adult tracts). The average percentage of White in the low-older-adult tracts is 49%, 24 percentage points lower than the average percentage of White in the high-older-adult tracts (i.e., 73%), a difference that is statistically significant (see column 4).

Among the census tracts in the high-older-adult category, what is their average racial and ethnic composition when disaggregated by the percentage of older adults living alone and in poverty? Columns 5 through 12 of Panel A in Table 2 address this question. The data show that census tracts considered to be high in older adults living alone and high in terms of older adults living in poverty tend to be more racially and ethnically diverse than those in the respective lower categories. For example, comparing columns 5 and 6 shows that, on average, 68% of the population in census tracts classified as high on the percentage of older adults living alone is White compared to 75% in tracts classified as low on the percentage of older adults living alone. Even more striking are the differences in columns 9 and 10. On average, 45% of the population in census tracts classified as high on the percentage of older adults living in poverty is White, compared to 77% in tracts classified as low on the percentage of older adults living in poverty.

Panel B of Table 2 examines the historical redlining score of census tracts by their older adult classifications. A score of 4.0 indicates that the entire census tract overlaps with an area marked as ‘D = Hazardous’ on the historical redlining map; scores that are 3.0 and above fall between the categories ‘C = Definitely Declining’ and ‘D = Hazardous’ [20]. Comparing the results in columns 1 and 2 shows that the percentages of tracts classified as high-older-adult tracts in the categories of redlining scores between 3 and less than 4 (i.e., 4.4%) and in scores equal to 4 (i.e., 0.5%) is lower than the respective percentages of tracts among low-older-adult tracts (i.e., 17% and 3%, respectively). However, the results in columns 5 through 12 show that when disaggregating the tracts classified as high-older-adult tracts into high and low categories based upon the percentage of the population alone and by poverty status, the high categories in these groupings are more likely than the low categories to be in historically redlined or declining areas. Examining columns 5 and 6, for example, reveals that 1% of tracts with a high percentage of older adults living alone are in the redlined category (i.e., score = 4), relative to 0.3% of tracts with a low percentage of older adults living alone. Comparing the same columns shows that 8% of tracts with a high percentage of older adults living alone have a redlining score between 3 and less than 4 compared to only 3.1% of tracts with a low percentage of adults living alone. The same patterns are found in the results when comparing census tracts with a high percentage of older adults living in poverty compared to those with a lower percentage of older adults living in poverty (see columns 9 and 10).

### 3.4. Social Capital of Older-Adult Neighborhoods

We now turn to an analysis comparing the social capital levels across older-adult neighborhoods as shown in Table 3. We use an overall measure of social capital, which is an index based upon three sub-indices gauging bonding, bridging, and linking capital defined above in Section 2. High values of the overall social capital index and these three sub-indices indicate high levels of social capital [26].

Columns 1 and 2 of Table 3 reveal that the average levels of social capital in high-older-adult neighborhoods are significantly greater than in low-adult neighborhoods, overall, and for the three sub-indices of bonding, bridging, and linking capital. However, once high-older-adult census tracts are disaggregated by the percentage of older adults living alone (columns 5 and 6) and by the percentage living in poverty (columns 9 and 10), it is clear that areas with greater percentages of older persons living alone (column 5) and in poverty (column 9) have significantly lower levels of social capital than tracts with lower percentages of older persons living alone (column 6) and in poverty (column 10), respectively. The one exception where there is no significant difference is among census tracts with high and low levels of persons living alone on the index gauging bridging capital.

### 3.5. Social Isolation of Older-Adult Neighborhoods

Table 4 presents analyses of the social isolation of older-adult neighborhoods overall and by the percentages of older adults living alone and below poverty. We measure social isolation with an index (see Section 2 above), where high values indicate more social isolation, and by the percentage of the population that is aged 65 and older. Columns 1 and 2 of Table 4 show that the average value of the social isolation index is significantly lower in census tracts classified as high-older-adult tracts relative to those classified as low-older-adult tracts (i.e., −0.14 versus 0.05). However, the average percentage of the population aged 65 and older is 25% in high-older-adult tracts compared to 13% in low-older-adult tracts. Taken together, these data suggest that although there are typically greater levels of the population aged 65 and older in high-older-adult tracts relative to low-older-adult tracts, it does not necessarily translate into social isolation.

How does social isolation vary among high-older-adult neighborhoods when disaggregating them by the percentages of older adults living alone and in poverty? Columns 5, 6, 9, and 10 of Table 4 address this question. Because the social isolation index is based on the percentage of the population living alone and on the poverty level, it is not surprising that census tracts classified as high on both indicators have greater levels of social isolation (see columns 5 and 9) compared to census tracts classified as low on these indicators (see columns 6 and 10), respectively. With respect to the percentage of the population aged 65 and older, it is not surprising that the differences between the high and low categories of these subgroups are similar, although they are statistically significant. The classification of high and low census tracts for the percentages of older adults living alone and in poverty is based only on census tracts with a high percentage of older adults.

### 3.6. Retail Food Provider Access and Quality of Older-Adult Neighborhoods

Does the access to retail food providers or the quality of retail food providers vary by the classification of neighborhoods by older adults? The data in Panels A and B of Table 5 address this question. Panel A focuses on the population’s access to retail food providers such as supermarkets or large grocery stores. The first row of Panel A focuses on “low-access tract at 1 and 10 miles”, which indicates the percentage of tracts whose residents live more than 1 mile (for urban areas) or more than 10 miles (for rural areas) from the nearest supermarket, supercenter, or large grocery store [22]. Columns 1 and 2 show that the percentage with low access is greater in high-older-adult tracts (i.e., 27%) than in low-older-adult tracts (i.e., 17%). Interestingly, the data in columns 5 and 6 reveal that those living in tracts classified as high on the percentage of older adults living alone are less likely to have low food access relative to those classified as low on the percentage of older adults living alone, although the difference is marginally significant (i.e., 23% versus 29%). Similarly, tracts classified as high on older adults living in poverty are less likely to have food access issues than those classified as low on older adults living in poverty (i.e., 7.7% versus 30%). In row 2, in Panel A of Table 5, a similar pattern of results emerges when examining “low-access tract at one-half (for urban areas) and more than 10 miles (for rural areas)”.

The third row of Panel A focuses on “low-access tract using vehicle access and at 20 miles”, which indicates the percentage of tracts whose residents live more than 20 miles, regardless of vehicle availability from the nearest supermarket, supercenter, or large grocery store [22]. Columns 1 and 2 show that the percentage with low access at that distance is greater in high-older-adult tracts (i.e., 20%) than in low-older-adult tracts (i.e., 15%). In contrast to the previous results, the data in columns 5 and 6 reveal that those living in tracts classified as high on the percentage of older adults living alone are more likely to have low food access at this 20-mile distance relative to those classified as low on the percentage of older adults living alone (i.e., 30% versus 16%). The difference in low food access for this distance, however, is not different between older adult tracts classified by their poverty status (see columns 9 and 10).

Panel B of Table 5 shows the data for our last food-related characteristic, the “modified retail food environment index” (mRFEI), which gauges the percentage of healthy food retailers in census tracts. Higher values indicate a higher number of healthy food retailers. Columns 1 and 2 show that, on average, census tracts classified as high-older-adult tracts have a greater percentage of healthy food retailers than those classified as a low-older-adult tracts (i.e., 12% versus 9%). Columns 5 and 6, however, show that there is little difference in the mRFEI average values between tracts classified by the percentage of adults living alone. On the other hand, columns 9 and 10 show that older adult tracts classified as having a high percentage of older adults living in poverty have lower average values of healthy food retailers than those classified as lower poverty tracts (i.e., 10% versus 12%).

### 3.7. Health Services of Older-Adult Neighborhoods

How does the access to health services vary by the classification of neighborhoods by older adults? The data in Panels A and B of Table 6 address this question. Panel A focuses on health services availability and spending. The first row of Panel A focuses on the mean number of health services within each classification of older-adult tracts. Columns 1 and 2 show that the mean number of health services in high-older-adult tracts is 12, which is significantly greater than the number in low-older-adult tracts (i.e., 8). The data in columns 5 and 6 reveal that those living in tracts classified as high on the percentage of older adults living alone are also significantly more likely to have a larger mean number of health services relative to those classified as low on the percentage of older adults living alone (i.e., 16 versus 10). However, tracts classified as high on older adults living in poverty are equivalent in their mean number of health services as those classified as low on older adults living in poverty (i.e., each with values of 12). In row 2, in Panel A of Table 6, the results show that the mean percentage of health services out of all businesses is significantly larger in high- versus low-older-adult census tracts (columns 1 and 2), and among high-older-adults census tracts, there is a greater mean percentage of health services in census tracts classified as having high levels of older persons living alone (i.e., columns 5 versus 6) and those in poverty (i.e., columns 9 versus 10). In row 3, in Panel A of Table 6, however, we see that the results are a bit different. While the mean value of spending on health services and insurance is higher in high versus low-older-adult census tracts (i.e., columns 1 and 2) and in census tracts with high and low levels of older adults living alone (i.e., columns 5 and 6), the amount spent is significantly lower in older adult tracts with high levels of poverty among older adults than in tracts with low levels of poverty (i.e., columns 9 and 10).

Panel B of Table 6 shows data for accessibility and distance to health services by older adult neighborhood classification. Interestingly, the data in columns 1 and 2 show that a significantly lower percentage of older-adult neighborhoods in the high category are considered medically underserved areas compared to the percentage of tracts in the low category of older adults. However, comparing columns 1 and 2 further down in Panel B shows that the average distances to all health services facilities are greater for high-older-adult tracts than for low-older-adult tracts. For example, the average distance to the emergency department in high-older-adult tracts is 3.8 compared to 2.5 in low-older-adult tracts. Considering high-older-adult tracts by the percentage of the older adult population living alone in columns 5 and 6, we see that neighborhoods classified as high with a larger share of older adults living alone are significantly more likely to be medically underserved areas relative to tracts considered as low on older adults living alone (i.e., 17% versus 4.3%). However, the average distances to all health services facilities are significantly smaller for tracts with greater shares of the population living alone than those with smaller shares. Considering high-older-adult tracts by the percentage of the older adult population living in poverty in columns 9 and 10, Table 6 shows that neighborhoods classified as high with a larger share of older adults living in poverty are more than nine times as likely to be medically underserved areas as tracts considered as low on older adults living in poverty (i.e., 34% versus 3.7%). However, the average distances to all health services facilities are significantly smaller for tracts with greater shares of the population living in poverty than those with smaller shares.

### 3.8. Summary of Key Findings

**Figure 3:**
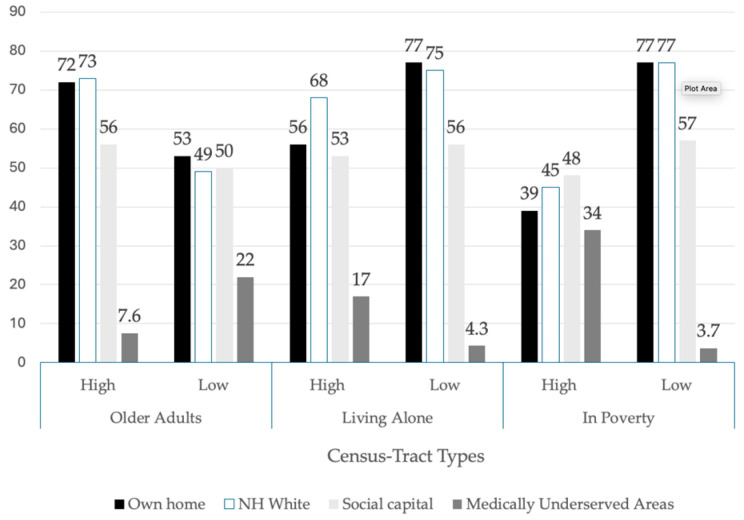
Key differences in characteristics between older-adult census-tract types.

To summarize the key findings of our ecological portrait of census tracts by their older-adult population and further disaggregated by the older-adult population: (a) living alone and (b) by poverty status, we highlight key comparisons in Figure 3.

As shown in the left panel of Figure 3, census tracts classified as “high” older-adult neighborhoods, on average, have higher homeownership rates, a non-Hispanic White population, and social capital and have lower percentages of medically underserved areas than census tracts classified as “low”-older-adult neighborhoods. However, when disaggregating older-adult census tracts by the percentage of the population living alone and in poverty, the pattern of these results shifts. Census tracts with older adult populations that have “high” levels of that population living alone, on average, have lower homeownership rates, percent non-Hispanic white population, social capital, and higher percentages of medically underserved areas than census tracts that have “low” levels of older-adult population living alone. The same pattern of differences is found between older-adult tracts classified as “high” in poverty and “low” in poverty. However, there are greater disparities in these characteristics. For example, the average homeownership rate in “high” poverty tracts is less than half of the rate in “low” poverty tracts. The percentage of medically underserved areas, on average, is nearly 10 times as high in “high” poverty tracts than in “low” poverty tracts.

## 4. Discussion

The main objective of our study was to provide an ecological portrait of the vulnerability of census tracts in NYS stratified by their older adult population. Our results suggest that, in general, neighborhoods with large shares of older adults overall and classified as high-older-adult neighborhoods in our study tend to fare well in terms of their demographic and economic characteristics, social fabric, and food and health services access. Our analyses revealed considerable spatial variation in the location of the high-older-adult census tracts spanning between rural areas in the Catskill and Adirondack regions of NYS to suburban areas within major NYS cities as well as locations in NYC primarily in non-central-business district locations. This spatial variation was similar to that found in Florida, the state with the largest share of the population aged 65 and older [8]. Our results on the higher levels of social capital and lower levels of medically underserved areas in older-adult neighborhoods are consistent with previous research that examined community social cohesion and medical access in communities in 20 countries [9].

Among those classified as having larger shares of older adults, we further disaggregated our analyses of census tracts by (1) the percentage living alone and (2) their poverty status, thereby acting as additional independent variables. Our results show that there are significant differences among these specific types of older-adult neighborhoods. Those with greater percentages of older adults living alone and in poverty face more vulnerabilities than their counterpart neighborhoods with lower percentages of older adults living alone and in poverty, respectively. Older-adult neighborhoods with larger shares of the population living alone and in poverty fare worse, living in areas with poorer socioeconomic status, lower social capital, more social isolation, and that are considered medically underserved. A distinction like this has not been made in previous research on older-adult neighborhoods [7,8,9].

Though the research in characterizing older adults’ neighborhoods is limited, our findings may be situated in the extant literature. For example, the finding that older adult neighborhoods are outside the centers of cities in NYS is similar to that reported in Calgary, Canada [27]. Furthermore, a study [28] in Colorado Springs (Colorado, CO, USA) finds that areas with large shares of affluent older adult households have better access to green space and other resources for active aging than those areas without. These income-based inequalities across neighborhoods are comparable to our findings with respect to the percentage of impoverished older populations in a tract.

The differences between high- and low-older-adult neighborhoods can be understood with several theoretical arguments. First, high-older-adult neighborhoods are the result of aging in place [29], which refers to older adults who have opted not to leave the dwellings in which they have spent most of their adult lives. These dwellings tend to be located in neighborhoods that have been developed for decades and are featured with resources, such as healthcare and food access. Importantly, such neighborhoods may attract older adults who are dissatisfied with their residences [30], which may in turn reinforce the concentration of older adults and demands for services in certain areas. Relatedly, older adults who do not move may enjoy stronger social networks and better social connections than those who move to other areas [31]. Second, Litwak and Longino [32] propose that older adults’ decision to relocate is driven by changes in health. Specifically, soon after retirement, older adults may move to areas where they can enjoy natural amenities or reactional events; however, when chronic disability hits, older adults may move to areas for ease of living (e.g., easy access to food or daily life). When preparing for severe diseases or disabilities, older adults may move to nursing homes. The changes in older adults’ health largely echo different needs in life, which may account for the differences between high and low-older-adult neighborhoods. Finally, based on the age-friendly communities model [4], older adults prefer to reside in areas that provide opportunities for older adults to stay socially engaged and/or active, freedom to move around neighborhoods, and easy access to healthcare providers. Such residential preferences may lead to the concentration of older adults in certain neighborhoods and the differences observed in this study.

Another important implication is that researchers should pay attention to the potential heterogeneities across neighborhoods with large shares of older adults. Specifically, the share of older adults may not be directly translated into neighborhood vulnerability; however, adding the layers of older adults living alone or in poverty to older-adult neighborhoods signifies a neighborhood’s vulnerability. This can be explained by the concept of intersectionality that emphasizes that individuals with multiple marginalizations are particularly vulnerable due to the oppressions from intersecting power domains [33,34]. Extending the intersectionality perspective to this study, our results indicate that advanced ages may not be a construct that influences neighborhood vulnerability; instead, older adults’ disability and socioeconomic disadvantage status are more relevant and tend to exert impacts on social inequalities, especially neighborhood vulnerability.

### 4.1. Policy Implications

From a policy perspective, our study suggests that several interventions should be considered so that older adults can age in neighborhoods that promote equity, physical accessibility, and social inclusiveness consistent with the principles established by the WHO [5]. Effective leadership and governance and the acquisition of resources stemming from political infrastructure are critical in promoting AFCC [6,35,36]. Given our findings about the inequalities that exist in socioeconomic resources among older-adult communities, with larger shares of the population living alone and in poverty, having a strong political infrastructure to garner resources from the state or federal government is critical. These could be in the form of grants or municipal funding, which has been used in interventions like transportation programs or recreation-based programs to promote health-based and social interaction programs [6]. Our findings demonstrated that older-adult communities face challenges with food access as well as access to quality health services. Programs that increase accessibility to transportation within these communities could significantly reduce these challenges and increase social networks and lower social isolation. Little research evaluates the access to mobility within older-adult communities, but studies have found it is essential to promoting AFCC [6,37,38].

### 4.2. Study Limitations

Our study is not without limitations. We are constrained to defining “neighborhoods” based on census tracts. While approximating homogenous areas, it is unclear whether older adults conceive of their neighborhoods in terms of census-based definitions of small geographic areas. Given that the data are not available below the census-tract level, this is a limitation that is faced by most ecological studies. We chose to present many different measures of vulnerability at the ecological level, including educational attainment, median household income, unemployment rate, and food access. This could be viewed as a limitation because of the large number of characteristics presented. However, the literature lacks agreement on the best set of characteristics to include in vulnerability indices to capture all aspects of vulnerability. A final limitation of our analyses is that they are descriptive in nature.

### 4.3. Directions for Future Research

Future research on older-adult neighborhood vulnerability should compare the indicators that we use here with indexes that combine the indicators because for older-adult communities, there may be different factors that are important in characterizing the vulnerability of those communities relative to those that are salient for communities of all ages. In addition, future research should examine how the factors presented here, alone or in conjunction with one another, would affect ecological- and individual-level environmental and health outcomes. Finally, much more research is needed on older-adult neighborhoods that go beyond NYS and examines all older-adult neighborhoods in the U.S. and other countries.

## 5. Conclusions

Given the projected growth in the older adult population in the coming decades, NYS policy makers should pay particular attention to the communities in which the most vulnerable segments of the older adult population live so that their residents can age in an environment that is equitable, physically accessible, and socially inclusive, thereby adhering to the principles established by WHO in maintaining age-friendly communities, underlying the UN Decade of Health Ageing 2021–2030 [5]. Vulnerability in older-adult communities is a shared phenomenon around the world, particularly as the population is aging rapidly worldwide. By comparing and identifying specific vulnerabilities in aging communities across countries, as our study has demonstrated in NYS, the global community can better learn what country-specific practices may work well in overcoming such barriers and ultimately work together towards creating policies to foster the healthy aging of communities.

## Figures and Tables

**Table 1:** Comparison of Socioeconomic and Demographic Characteristics by Older Adult Classifications of Census Tracts.

Mean/(SD)	Percentage of Older Adults (65+)					Percentage of Older Adults Living Alone					Percentage of Older Adults Living Below Poverty Line			
Characteristic	High (1)	Low (2)	95% CI (3)	*p*-Value (4)		High (5)	Low (6)	95% CI (7)	*p*-Value (8)		High (9)	Low (10)	95% CI (11)	*p*-Value (12)
Education BA or higher (%)	40 (18)	33 (19)	5.7, 8.1	<0.001		41 (21)	39 (17)	−0.50, 4.6	0.11		34 (17)	41 (18)	−10, −4.0	<0.001
*N*	*1213*	*3636*				*316*	*897*				*156*	*1057*		
Median income	40,223 (16,122)	35,330 (16,970)	3829, 5958	<0.001		40,236 (19,640)	40,218 (14,701)	−2365, 2401	>0.9		29,160 (15,007)	41,839 (15,643)	−15,246, −10,113	<0.001
*N*	*1208*	*3628*				*314*	*894*				*154*	*1054*		
Own house (%)	72 (22)	53 (29)	17, 21	<0.001		56 (24)	77 (18)	−25, −19	<0.001		39 (26)	77 (16)	−42, −33	<0.001
*N*	*1212*	*3616*				*316*	*896*				*155*	*1057*		
Older adults with disabilities (%)	32 (11)	34 (13)	−3.3, −1.8	<0.001		37 (12)	30 (10)	5.2, 8.2	<0.001		42 (14)	30 (9)	9.4, 14	<0.001
*N*	*1213*	*3598*				*316*	*897*				*156*	*1057*		
Unemployment Rate	2.70 (1.66)	3.81 (2.92)	−1.2, −0.98	<0.001		2.84 (1.83)	2.66 (1.60)	−0.05, 0.41	0.12		3.30 (2.10)	2.62 (1.57)	0.34, 1.0	<0.001
*N*	*1213*	*3639*				*316*	*897*				*156*	*1057*		

Source: Tabulations of American Community Survey 2019 5-year data. Notes: 1. Values in cells are mean (SD). 2. The threshold for high/low is 75th percentile. 3. *p*-values generated from Welch Two Sample *t*-test; *p*-values less than 0.05 are statistically significant.

**Table 2:** Comparison of Racial/Ethnic and Redlining Characteristics by Older Adult Classifications of Census Tracts.

	Percentage of Older Adults (65+)					Percentage of Older Adults Living Alone					Percentage of Older Adults Living Below Poverty Line			
	High (1)	Low (2)	95% CI (3)	*p*-Value (4)		High (5)	Low (6)	95% CI (7)	*p*-Value (8)		High (9)	Low (10)	95% CI (11)	*p*-Value (12)
Panel A. Racial/ethnic composition—percentages														
NH Black Racial composition	8 (18)	18 (24)	−11, −8.5	<0.001		11 (19)	7 (18)	1.8, 6.7	<0.001		17 (24)	7 (17)	6.6, 14	<0.001
NH White Racial composition	73 (27)	49 (34)	22, 25	<0.001		68 (27)	75 (27)	−11, −3.8	<0.001		45 (31)	77 (24)	−38, −27	<0.001
Asian Racial composition	8 (13)	9 (13)	−1.9, −0.19	0.017		7 (10)	8 (14)	−2.3, 0.68	0.3		16 (22)	6 (11)	6.5, 14	<0.001
Hispanic Racial composition	9 (11)	21 (21)	−13, −11	<0.001		11 (13)	8 (10)	1.5, 4.7	<0.001		19 (19)	8 (8)	8.0, 14	<0.001
*N (census tracts)*	*1213*	*3640*				*316*	*897*				*156*	*1057*		
Source: Tabulations of American Community Survey 2019 5-year data														
Panel B. Redlining score				<0.001					<0.001					<0.001
0 <= Average redlining < 3	1014 (95%)	2620 (80%)				260 (91%)	755 (97%)				119 (82%)	895 (97%)		
3 <= Average redlining < 4	47 (4.4%)	563 (17%)				23 (8.0%)	24 (3.1%)				25 (17%)	22 (2.4%)		
Average redlining score = 4	5 (0.5%)	100 (3.0%)				3 (1.0%)	2 (0.3%)				2 (1.4%)	3 (0.3%)		
*N (census tracts)*	*1066*	*3283*				*286*	*780*				*146*	*920*		

Source: Mapping Inequality redlining maps—https://dsl.richmond.edu/panorama/redlining/ (accessed on 30 September 2024). Notes: 1. Values in cells are n (%) or mean (SD). 2. The threshold for high/low is 75th percentile. 3. For redlining score, *p*-values generated from Chi-square test; *p*-values less than 0.05 are statistically significant. 4. For racial/ethnic composition, *p*-values generated from Welch Two Sample *t*-test; *p*-values less than 0.05 are statistically significant.

**Table 3:** Comparison of Social Capital by Older Adult Classifications of Census Tracts.

	Percentage of Older Adults (65+)					Percentage of Older Adults Living Alone					Percentage of Older Adults Living Below Poverty Line			
	High (1)	Low (2)	95% CI (3)	*p*-Value (4)		High (5)	Low (6)	95% CI (7)	*p*-Value (8)		High (9)	Low (10)	95% CI (11)	*p*-Value (12)
Social Capital	0.56 (0.09)	0.50 (0.10)	0.05, 0.06	<0.001		0.53 (0.08)	0.56 (0.09)	−0.04, −0.02	<0.001		0.48 (0.09)	0.57 (0.08)	−0.11, −0.08	<0.001
Bonding	0.51 (0.09)	0.50 (0.10)	0.01, 0.02	<0.001		0.47 (0.09)	0.52 (0.09)	−0.06, −0.04	<0.001		0.41 (0.10)	0.52 (0.08)	−0.13, −0.10	<0.001
Bridging	0.52 (0.19)	0.42 (0.20)	0.09, 0.11	<0.001		0.50 (0.18)	0.52 (0.19)	−0.04, 0.01	0.14		0.41 (0.21)	0.53 (0.18)	−0.15, −0.08	<0.001
Linking	0.64 (0.10)	0.59 (0.12)	0.05, 0.06	<0.001		0.63 (0.11)	0.65 (0.10)	−0.04, −0.01	0.002		0.60 (0.12)	0.65 (0.10)	−0.07, −0.03	<0.001
*N*	*1213*	*3640*				*316*	*897*				*156*	*1057*		

Source: Fraser, Page-Tan, and Aldrich 2022. Notes: 1. Social Capital: average of three subindices—bonding, bridging, and linking capital. Positive values indicate greater social capital. a. Bonding Capital: uses nine indicators to gauge how similar people are in a community in terms of age, race, class, gender, language, and community capacity; high values indicate high levels of homogeneity, which is thought to have strong bonds. b. Bridging Capital: uses six indicators of “membership in associations that can bridge different parts of society” (Fraser et al. 2022: 10) and include the number of unions and the following types of organizations: religious, civic, social advocacy, and charitable organizations—all per 10,000 residents; also includes the percentage of residents participating in a fraternal order (at the county level). c. Linking Capital: uses five indicators that examine the percentage of: voting eligible citizens; and local, state, and federal government employees per capita. The percentage of residents who attended a political rally, speech or organized protest in the past year at the county level was also included. 2. Values in cells are mean (SD). 3. The threshold for high/low is 75th percentile. 4. *p*-values generated from Welch Two Sample *t*-test; *p*-values less than 0.05 are statistically significant.

**Table 4:** Comparison of Social Isolation by Older Adult Classifications of Census Tracts.

	Percentage of Older Adults (65+)					Percentage of Older Adults Living Alone					Percentage of Older Adults Living Below Poverty Line			
Social Isolation	High (1)	Low (2)	95% CI (3)	*p*-Value (4)		High (5)	Low (6)	95% CI (7)	*p*-Value (8)		High (9)	Low (10)	95% CI (11)	*p*-Value (12)
Social isolation index	−0.14 (0.60)	0.05 (0.68)	−0.23, −0.15	<0.001		0.43 (0.59)	−0.35 (0.45)	0.71, 0.85	<0.001		0.74 (0.71)	−0.27 (0.46)	0.90, 1.1	<0.001
N (census tracts)	1213	3598				316	897				156	1057		
Percentage 65+	25 (7)	13 (4)	11, 12	<0.001		25.8 (8.6)	24.4(5.7)	0.33, 2.4	0.01		26.0 (7.4)	24.6 (6.4)	0.20, 2.7	0.023
*N (census tracts)*	*1213*	*3640*				*316*	*897*				*156*	*1057*		

Source: Tabulations of American Community Survey 2019 5-year data. Notes: 1. Social isolation index is defined here—https://assets.americashealthrankings.org/app/uploads/ahrsenior18-finalv1.pdf (accessed on 30 September 2024), which says on p. 159 that it is the percentile of the mean z-scores for six risk factors of social isolation in adults aged 65 and older (poverty; living alone; divorced, separated, or widowed; never married; disability; independent living difficulty). 2. Values in cells are mean (SD). 3. The threshold for high/low is 75th percentile. 4. *p*-values generated from Welch Two Sample *t*-test; *p*-values less than 0.05 are statistically significant.

**Table 5:** Comparison of Food Access Characteristics by Older Adult Classifications of Census Tracts.

	Percentage of Older Adults (65+)					Percentage of Older Adults Living Alone					Percentage of Older Adults Living Below Poverty Line			
	High (1)	Low (2)	95% CI (3)	*p*-Value (4)		High (5)	Low (6)	95% CI (7)	*p*-Value (8)		High (9)	Low (10)	95% CI (11)	*p*-Value (12)
Panel A. Food Access														
Low-access tract at 1 and 10 miles	328 (27%)	608 (17%)	7.5%, 13%	<0.001		72 (23%)	256 (29%)	−11%, −0.05%	0.057		12 (7.7%)	316 (30%)	−28%, −17%	<0.001
Low-access tract at one-half and 10 miles	573 (47%)	1395 (38%)	5.6%, 12%	<0.001		153 (48%)	420 (47%)	−5.0%, 8.2%	0.7		33 (21%)	540 (51%)	−37%, −22%	<0.001
Low-access tract using vehicle access and at 20 miles	242 (20%)	543 (15%)	2.4%, 7.6%	<0.001		96 (30%)	146 (16%)	8.3%, 20%	<0.001		27 (17%)	215 (20%)	−9.8%, 3.7%	0.4
*N (census tracts)*	*1213*	*3637*				*316*	*897*				*156*	*1057*		
Source: USDA data on food access (see https://www.ers.usda.gov/data-products/food-access-research-atlas/documentation/ (accessed on 30 September 2024) for definitions)														
Panel B. Quality of food retailers														
Modified Retail Food Environment Index (%)—mRFEI	12 (13)	9 (9)	2.1, 3.9	<0.001		11 (11)	13 (13)	−3.2, 0.21	0.086		10 (10)	12 (13)	−4.3, −0.44	0.017
*N (census tracts)*	*990*	*3146*				*261*	*729*				*131*	*859*		

Source: Amin, Baddruddoza, and McCluskey 2021—https://www.ncbi.nlm.nih.gov/pmc/articles/PMC7564312/ (accessed on 30 September 2024). Notes: 1. Values in cells are n (%) or mean (SD). 2. The threshold for high/low is 75th percentile. 3. *p*-values generated from Welch Two Sample *t*-test. For Low-income Low-access at 1 and 10 miles n (%) and Percentage of Older Adults Living below Poverty Line, *p*-values generated from Fishers’ exact test; *p*-values less than 0.05 are statistically significant.

**Table 6:** Comparison of Health Services by Older Adult Classifications of Census Tracts.

	Percentage of Older Adults (65+)					Percentage of Older Adults Living Alone					Percentage of Older Adults Living Below Poverty Line			
	High (1)	Low (2)	95% CI (3)	*p*-Value (4)		High (5)	Low (6)	95% CI (7)	*p*-Value (8)		High (9)	Low (10)	95% CI (11)	*p*-Value (12)
Panel A. Health Services Availability and Spending														
Number of health services (mean)	12 (8)	8 (14)	2.8, 5.2	<0.001		16 (22)	10 (16)		<0.001		12 (14)	12 (18)		0.9
Percentage of health services out of all businesses (mean)	7.4 (7.1)	5.3 (5.8)	1.6, 2.5	<0.001		9 (7)	7 (7)		<0.001		9 (8)	7 (7)		0.011
Total spending on health services including insurance (in millions USD$) (mean)	13.24 (10.2)	10.47 (8.6)	2.1, 3.5	<0.001		14.88 (13.9)	12.64 (8.4)		0.011		9.52 (9.4)	13.83 (10.2)		<0.001
*N (census tracts)*	*1066*	*3239*				*286*	*780*				*146*	*920*		
Source: ESRI Business Summary Data														
Panel B. Accessibility and Distance to Health Services														
Medically Underserved Areas														
Yes (%)	7.6% (92)	22% (798)	−16%, −12%	<0.001		17% (54)	4.3% (38)	8.3%, 17%	<0.001		34% (53)	3.7% (39)	22%, 38%	<0.001
Distance in miles to the nearest:														
Emergency department	3.8 (4.4)	2.5 (3.1)	1.1, 1.6	<0.001		2.3 (2.7)	4.3 (4.7)	−2.5, −1.6	<0.001		1.3 (1.5)	4.2 (4.5)	−3.3, −2.6	<0.001
Medical-surgical ICU	4.1 (4.9)	2.6 (3.4)	1.2, 1.8	<0.001		2.3 (2.7)	4.8 (5.3)	−2.9, −2.0	<0.001		1.3 (1.7)	4.6 (5.1)	−3.7, −2.9	<0.001
Designated trauma center	11 (13)	7 (10)	3.9, 5.6	<0.001		8 (11)	12 (14)	−6.1, −3.0	<0.001		4 (7)	12 (14)	−9.4, −6.5	<0.001
Health clinic	4.4 (4.9)	2.5 (3.7)	1.7, 2.3	<0.001		3.0 (4.2)	5.0 (5.1)	−2.6, −1.5	<0.001		1.4 (2.3)	4.9 (5.1)	−4.0, −3.0	<0.001
Hospital with alcohol and drug abuse inpatient care	6.4 (7.4)	4.1 (5.7)	1.9, 2.8	<0.001		4 (5)	7 (8)	−3.9, −2.3	<0.001		2 (3)	7 (8)	−5.5, −4.1	<0.001
*N (census tracts)*	*1209*	*3632*				*316*	*893*				*156*	*1053*		

Source: Social Determinants of Health Database, 2019—https://www.ahrq.gov/sdoh/data-analytics/sdoh-data.html (accessed on 30 September 2024). Notes: 1. Values in cells are mean (SD) or where noted % (n). 2. The threshold for high/low is 75th percentile. 3. *p*-values generated from Welch Two Sample *t*-test; *p*-values less than 0.05 are statistically significant.

## Data Availability

The demographic and socioeconomic data from the 2019, 5-year release of the ACS are available at: https://www.census.gov/data/developers/data-sets/acs-5year.2019.html#list-tab-1806015614. The historical redlining maps used to calculate redlining scores are available at: https://dsl.richmond.edu/panorama/redlining/ (accessed on 30 September 2024). The USDA 2019 Food Access Research Atlas Data are available at: https://www.ers.usda.gov/data-products/food-access-research-atlas/ (accessed on 30 September 2024). The mRFEI data are available at: https://stacks.cdc.gov/view/cdc/61367 (accessed on 30 September 2024). ESRI business data are available from ArcGIS Pro: https://pro.arcgis.com/en/pro-app/latest/help/analysis/business-analyst/data-overview.htm (accessed on 30 September 2024). The Social Determinants of Health database was compiled by the Agency for Healthcare Research and Quality (AHRQ) and is available at: https://www.ahrq.gov/sdoh/data-analytics/sdoh-data.html (accessed on 30 September 2024). The social capital index data are available at: https://doi.org/10.7910/DVN/OSVCRC (accessed on 30 September 2024). The social isolation index data are available at: https://assets.americashealthrankings.org/app/uploads/ahrsenior18-finalv1.pdf (accessed on 30 September 2024).
